# Effects of Intraoperative Ketamine on Post-Operative Recovery in Obstructive Sleep Apnea Patients: A Case-Control Study

**DOI:** 10.7759/cureus.8893

**Published:** 2020-06-28

**Authors:** Nicole M Schreiner, Hari Kalagara, Charity J Morgan, Ayesha Bryant, David L Benz, Timothy J Ness, Promil Kukreja, Peter Nagi

**Affiliations:** 1 Anesthesiology and Perioperative Medicine, University of Alabama at Birmingham, Birmingham, USA; 2 Biostatistics, University of Alabama at Birmingham, Birmingham, USA

**Keywords:** obstructive sleep apnea, post-operative opioids, peri-operative analgesia, perioperative pain management, body mass index

## Abstract

Objective

To evaluate the post-operative outcomes of patients with obstructive sleep apnea (OSA) given intraoperative ketamine.

Design: case-control study

A total of 574 patients (287 received ketamine and 287 were matched controls) diagnosed with OSA and body mass index (BMI) > 30 who received general anesthesia were included in this study. Patients given intraoperative ketamine were matched (1:1) with those who did not receive ketamine for age, gender, BMI, ethnicity, anesthesia time, intraoperative fentanyl dose, ketamine dose, and surgery type. A sub-analysis was performed based on the dose of ketamine administered and also on the surgery type. Measured outcomes include post-operative pain scores, post-operative opioid requirements, respiratory status, oxygen use, and duration post-operatively.

Results

Intraoperative ketamine use did not decrease pain scores or post-operative opioid use when compared with the control (no intraoperative ketamine) group. Patients who received high-dose ketamine had significantly higher post-operative pain scores (p=0.048) while in the post-anesthesia care unit (PACU) and required supplemental oxygen for a longer period of time (p = 0.030), pain scores were not significant for patients who underwent orthopedic/spine procedures (p = 0.074), and high-dose ketamine group patients who underwent orthopedic/spine surgery required significantly more opioids in the PACU (p = 0.031). Among patients who received low-dose ketamine, those who underwent head, ear, nose, and throat surgery required significantly more opioids in PACU (p = 0.022).

Conclusions

Low-dose intraoperative ketamine did not decrease pain scores or post-operative opioid use significantly and did not improve standard respiratory recovery parameters for OSA patients after surgery. Neither low- nor high-dose ketamine demonstrated the anticipated benefits of low pain scores and reduced post-operative opioid use. These outcomes will differ depending on the surgery type and dose of ketamine used.

## Introduction

Obstructive sleep apnea (OSA) affects up to 25% of elective surgical patients [1], many of whom are undiagnosed. OSA often accompanies obesity and is an independent risk factor for morbidity and mortality following anesthesia [2]. The STOP-Bang questionnaire is used to screen patients for the probability of OSA [3]. Despite many published trials, the role of ketamine as a component of perioperative analgesia is unclear [4]. Some of the difficulties encountered in these patients include difficult mask ventilation secondary to redundant soft tissue, decreased respiratory reserve leading to rapid desaturation, blood sugar issues secondary to diabetes, and uncontrolled hypertension, to name a few [5-7]. An additional hurdle includes post-operative pain management, as OSA patients demonstrate decreased pain tolerance post-operatively. This decreased pain tolerance, combined with baseline hypopnea and increased sensitivity to respiratory depression by opioids, makes their post-operative course especially dangerous and complicated [8-9].

Intravenous (IV) opioids are the most commonly used drugs for pain control in the post-anesthesia care unit (PACU). These analgesics work by binding to mu opioid receptors, which are expressed in both the central nervous system and the peripheral nervous system. Binding inhibits nociceptive signaling, leading to decreased pain sensation. However, these receptors also inhibit autonomic circuits, which help regulate respiratory and cardiac function, leading to the respiratory depression and hypotension often seen following opioid administration [6]. Given these effects and the vulnerability of the OSA population, it is important that anesthesiologists work to find ways to minimize post-operative opioid needs in the OSA patient population.

Ketamine is a dissociative anesthetic that works by binding to and antagonizing NMDA receptors. In a study by Eikermann et al., ketamine has been shown to activate respiratory muscles despite loss of consciousness during general anesthesia [10]. Additionally, a systematic review by Laskowski in 2011 found that ketamine lowered pain scores and opioid requirements post-operatively when given with general anesthesia [11]. In a paper by Gupta et al., researchers showed that ketamine enhances opioid-induced phosphorylation of signaling molecules and sped up resensitization of opioid-induced signaling, leading to enhanced analgesic effects and increased duration of action of opioids [12]. However, no studies to date have looked specifically at the use of ketamine in obese patients with OSA. We hypothesized that the use of intraoperative ketamine in OSA patients would decrease post-operative pain scores, post-operative opioid requirements, and respiratory depression in the PACU.

## Materials and methods

Single-institute patients undergoing general anesthesia with a current diagnosis of OSA who received general anesthesia between January 1, 2016, and January 6, 2018, were 18-100 years of age, had an American Society of Anesthesiologists Score (ASA) 1-4, and had a body mass index (BMI) of >30 were included in this study. Obstetric patients and patients who underwent a cardioversion and sleep endoscopy procedures were excluded. For patients who had multiple procedures during the study period, only the first procedure was included. All study activities were approved by the University of Alabama at Birmingham’s Institutional Review Board.

The primary endpoints for this study were average PACU pain score measured using visual analog scores (VAS, range of 0-10) and total opioid use in PACU (measured in oral morphine equivalent [OME] units). Using the Medical Administration Record from the electronic medical record in conjunction with the charted PACU admission and discharge times, the amount of opioids given in PACU was obtained and converted to OMEs using the standard CDC conversion table (https://www.cms.gov/Medicare/Prescription-Drug-Coverage/PrescriptionDrugCovContra/Downloads/Opioid-Morphine-EQ-Conversion-Factors-Aug-2017.pdf).

Secondary endpoints included time until the patient first requested pain medication, total time spent in PACU (or time until the patient was ready for discharge from the PACU), duration for which supplemental oxygen was required post-operatively, number of documented desaturation events (defined as SpO_2_ < 92%), and the incidence of post-operative nausea/vomiting and delirium. Time to first requested pain medication and time of required supplemental oxygen were censored at 240 minutes.

Matching

Patients who received ketamine were matched (1:1) with non-ketamine patients on age (within 10 years), gender, BMI (within five points), race (black, white, or other), anesthesia time (within 30 minutes), intraoperative fentanyl dose (within 50 mcg), ketamine dose, and surgery type (musculoskeletal/spine, head/neck, gastrointestinal/genitourinary, vascular, cardiac, craniotomies/spinal cord, or superficial/minor). Patients receiving ketamine were further stratified according to ketamine dosing as either high-dose ketamine (≥ 50 mg) or low-dose (< 50 mg).

Statistical analysis

Analysis was performed using SAS v. 9.4 (SAS Inc., Cary, NC, USA). Continuous data were summarized as mean and standard error (SE), categorical data were summarized as frequency and percentages, and time-to-event data were summarized as median and interquartile range (IQR). Two sample t-tests (for continuous variables), chi-square tests (for categorical variables), and log-rank tests (for time-to-event variables) were used to compare patients who received ketamine intraoperatively with the matched controls. These analyses were conducted on the entire sample as well as after stratifying by ketamine dose and surgery type. A p-value of <0.05 was considered statistically significant.

## Results

A total of 574 patients were analyzed (287 received ketamine and 287 were matched controls). Demographics and clinical characteristics for the matched sample are shown in Table 1.

**Table 1:** Demographics and clinical characteristics *p < 0.05. SE, standard error; BMI, body mass index

Characteristics	Ketamine (n = 287)	No Ketamine (n = 287)	p-Value*
Age, mean (SE)	57.7 (0.61)	58.7 (0.59)	0.270
Gender, n (%)			0.999
Female	141 (49.1%)	141 (49.1%)	
Male	146 (50.9%)	146 (50.9%)	
Race, n (%)			0.999
Black	76 (26.5%)	76 (26.5%)	
White	203 (70.7%)	203 (70.7%)	
Other	8 (2.8%)	8 (2.8%)	
BMI, mean (SE)	37.87 (0.38)	37.62 (0.38)	0.648
Anesthesia minutes, mean (SE)	168.30 (5.68)	168.61 (5.51)	0.969
Total fentanyl dose, mean (SE)	160.80 (7.26)	161.19 (6.91)	0.969
Surgery category, n (%)			0.999
Gastrointestinal/genitourinary	85 (29.6%)	85 (29.6%)	
Cardiac	9 (3.1%)	9 (3.1%)	
Head/neck	24 (8.4%)	24 (8. %)	
Musculoskeletal/spine	80 (27.87%)	80 (27.87%)	
Superficial/minor	85 (29.62%)	85 (29.62%)	
Vascular	4 (1.05%)	4 (1.05%)	
Chronic opioid use, n (%)			0.754
Yes	23 (8.01%)	21 (7.32%)	
No	264 (91.99%)	266 (92.68%)	
Ketamine dose, mean (SE)	42.43 (1.80)	----	----
Ketamine dose group, n (%)			
Low (<50 mg), n (%)	169 (58.89%)	----	----
High (≥50 mg), n (%)	118 (41.11%)	----	----

The incidence of chronic opioid use, as determined by ICD-9 and ICD-10 coding, did not significantly differ between the ketamine and no ketamine groups (8.01% vs. 7.32%, respectively; p = 0.754). Furthermore, among ketamine patients, the incidence of chronic opioid use did not significantly differ according to ketamine dose (5.92% for low dose vs. 11.02% for high dose; p = 0.117).

Ketamine patients did not significantly differ from non-ketamine patients in pain scores, OME requirements, adverse events, or time until ready for PACU discharge (Table 2).

**Table 2:** Study endpoints *p < 0.05. PACU, post-anesthesia care unit; SE, standard error; OME, oral morphine equivalent; IQR, interquartile range

Endpoint	Ketamine (n = 287)	No Ketamine (n = 287)	p-Value*
Average pain scores in PACU, mean (SE)	3.10 (0.20)	2.84 (0.18)	0.332
Total OME requirements in PACU, mean (SE)	7.15 (0.55)	6.41 (0.51)	0.328
Time to first opioid dose in PACU (minutes), median (IQR)	34 (14, 240+)	35 (15, 240+)	0.901
Time to wean to room air (minutes), median (IQR)	240 (48, 240+)	155 (34, 240+)	0.038
Desaturation events, mean (SE)	0.34 (0.07)	0.38 (0.08)	0.750
Adverse events			
Nausea or vomiting, n (%)	7 (2.44%)	7 (2.44%)	1.000
Dysphoria, n (%)	4 (1.39%)	1 (0.35%)	0.178
Total PACU time, mean (SE)	101.08 (3.72)	96.94 (3.56)	0.422

**Figure 1:**
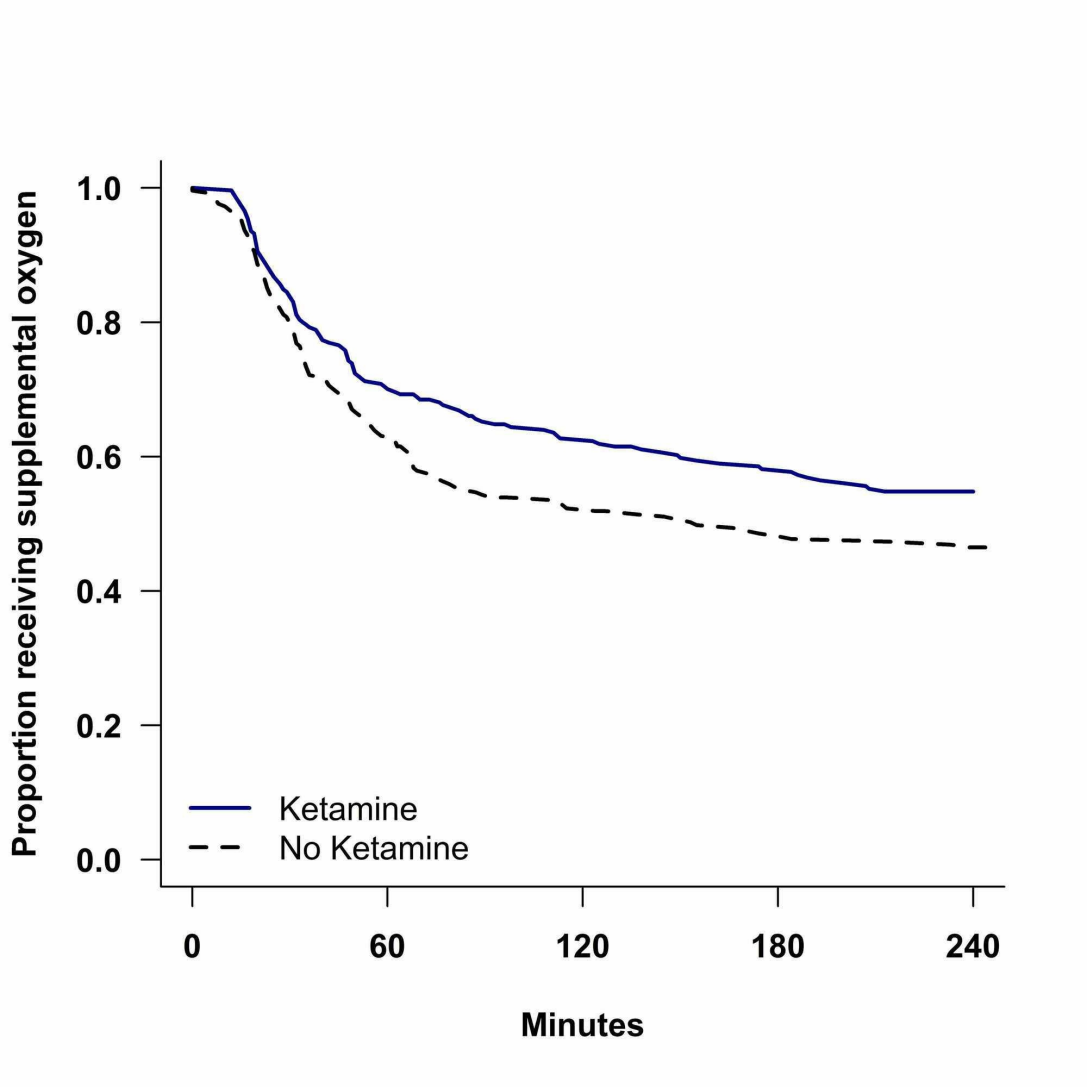
Time to wean to room air

Ketamine patients had significantly longer times to wean to room air than their matched controls (p = 0.038). Only 48.43% of ketamine patients had weaned to room air by 120 minutes post-operatively compared with 56.45% of non-ketamine patients. Kaplan-Meier estimates for the time to wean to room air for the ketamine and non-ketamine groups are shown in Figure 1.

Results by dose

High-Dose Ketamine vs. Control

Among the ketamine patients, 118 (41%) received high-dose ketamine and 169 (59%) received low-dose ketamine. When comparing the patients who received high-dose ketamine to their matched controls, the increased time to wean to room air was again noted in the ketamine group (p = 0.038). Additionally, the high-dose ketamine patients reported a higher average (SE) pain score, 3.98 (0.32), on arrival to PACU compared with their matched controls, who reported an average (SE) pain score of 3.25 (0.27; p = 0.048). Despite this difference, these patients did not require a significantly higher amount of opioids to control their pain (p = 0.105) or ask for medication sooner than the non-ketamine patients (p = 0.884). There was no difference noted in the incidence of adverse events or desaturation events between the high-dose ketamine and matched control group, or the time until ready for PACU discharge (p > 0.20 for all).

**Figure 2:**
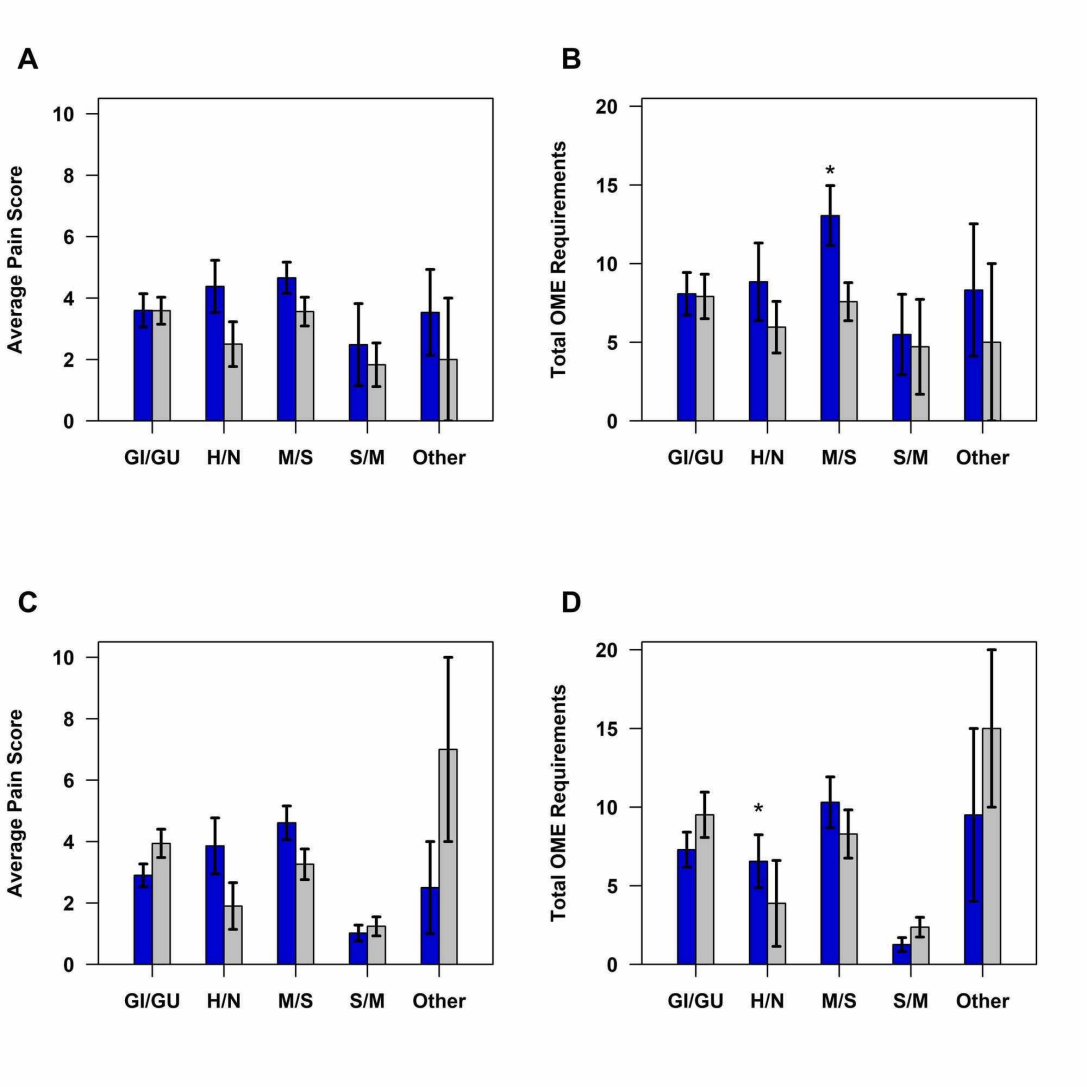
(A) Average pain scores for high-dose ketamine patients and their matched controls. (B) Total OME requirements for high-dose ketamine patients and their matched controls. (C) Average pain scores for low-dose ketamine patients and their matched controls. (D) Total OME requirements for low-dose ketamine patients and their matched controls. Blue bar indicates ketamine, and gray bar indicates no ketamine. *p < 0.05. GI/GU, gastrointestinal/genitourinary; H/N, head/neck; M/S, musculoskeletal/spine; S/M, superficial/minor; Other, vascular and cardiac; OME, oral morphine equivalent

When the patients who received high-dose ketamine were further stratified by surgical category, the patients who received high-dose ketamine reported higher pain scores on arrival to PACU for the head/neck (4.38 vs. 2.50; p = 0.106) and musculoskeletal/spine (4.66 vs. 3.56; p = 0.074) categories (Figure 2A); however, these results did not achieve statistical significance. In regards to total PACU OME requirements, the high-dose ketamine group required more opioids in PACU for musculoskeletal/spine procedures (13.05 vs. 7.58; p = 0.031) (Figure 2B).

Low-Dose Ketamine vs. Control

When comparing patients who received low-dose ketamine with their matched controls, there was no noted difference in the time to wean to room air (p = 0.246) or time to first opioid (p = 0.887). There was no significant difference in pain scores on arrival to PACU, OME requirements post-operatively, adverse events, or time until ready for PACU discharge (p > 0.20 for all).

When further divided by surgical category, there was again a difference noted in pain scores on arrival to PACU, with the head/neck (3.86 vs 1.90; p = 0.114) and musculoskeletal/spine (4.61 vs 3.26; p = 0.099) procedure patients who received ketamine reporting higher pain scores than their matched controls (Figure 2C). However, the results were not statistically significant. In regard to OME requirements, the head/neck patients who received low-dose ketamine required significantly more opioids than their matched controls (6.55 vs 3.88; p = 0.022) (Figure 2D).

Results by surgical category

**Figure 3:**
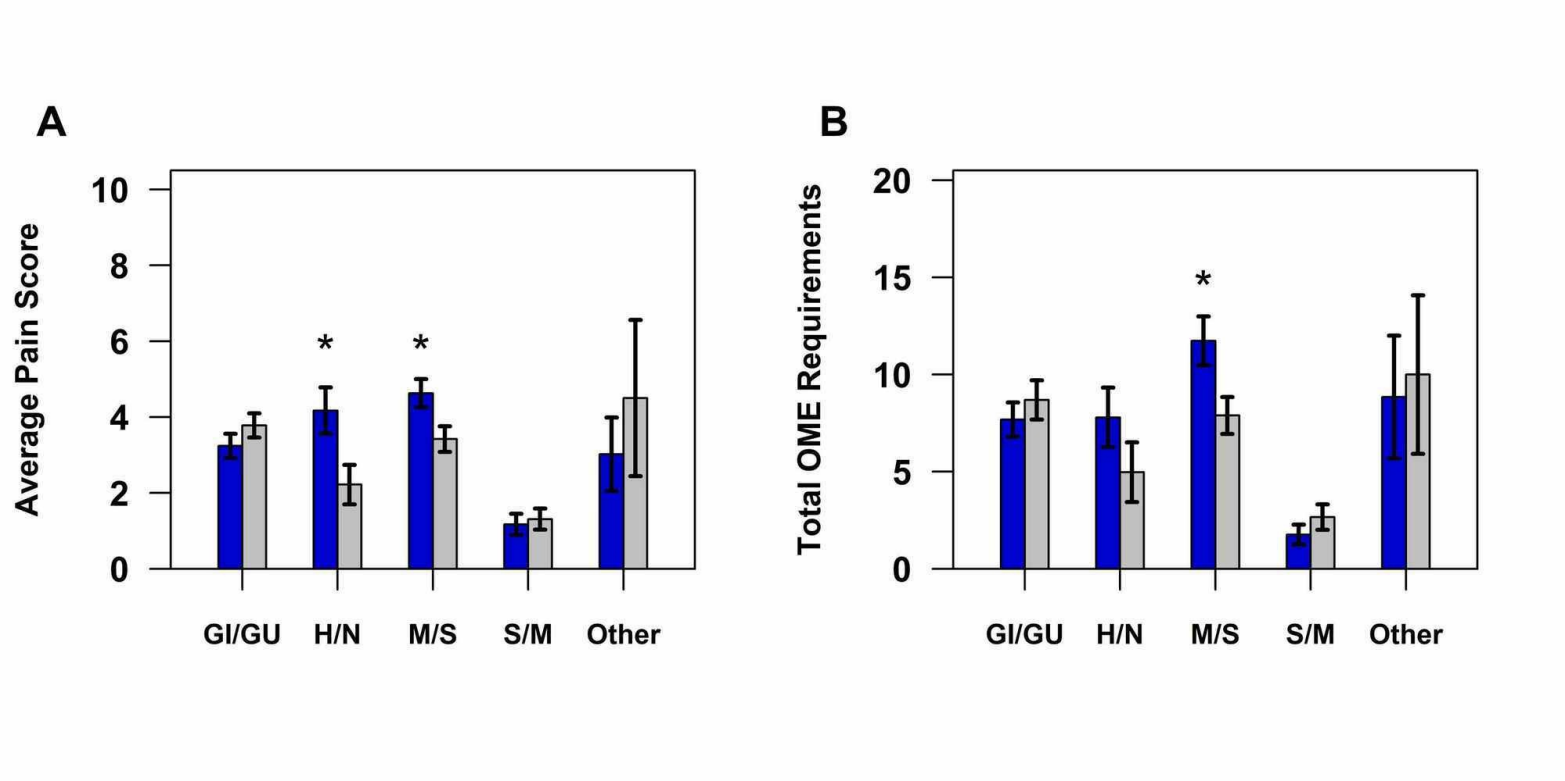
(A) Average pain scores and (B) total OMEs by surgery type. Blue bar indicates ketamine, and gray bar indicates no ketamine. *p < 0.05. GI/GU, gastrointestinal/genitourinary; H/N, head/neck; M/S, musculoskeletal/spine; S/M, superficial/minor; Other, vascular and cardiac; OME, oral morphine equivalent

Due to the small sample sizes, the cardiac (n = 18) and vascular (n = 8) patients were combined. Ketamine patients had significantly higher pain scores than the non-ketamine patients for both head/neck surgeries (p = 0.024) and musculoskeletal/spine surgeries (p = 0.013) (Figure 3A).

For the musculoskeletal/spine surgeries, ketamine patients also required significantly more OMEs (p = 0.024) (Figure 3B). No other significant differences in pain scores or OME requirements were observed for any of the remaining surgical categories (Table 3).

**Table 3:** Primary study endpoints by surgery type *p < 0.05 PACU, post-anesthesia care unit; SE, standard error; OME, oral morphine equivalent

Endpoint	Surgery Type	Ketamine	No Ketamine	p-Value*
Average pain scores in PACU, mean (SE)	Gastrointestinal/genitourinary (n = 170)	3.24 (0.32)	3.78 (0.32)	0.208
	Head/neck (n = 48)	4.17 (0.61)	2.22 (0.52)	0.024
	Musculoskeletal/spine (n = 160)	4.63 (0.37)	3.42 (0.34)	0.013
	Superficial/minor (n = 170)	1.17 (0.28)	1.31 (0.28)	0.676
	Cardiac, vascular (n = 26)	3.02 (0.97)	4.50 (2.06)	0.469
Total OME requirements in PACU, mean (SE)	Gastrointestinal/genitourinary (n = 170)	7.68 (0.88)	8.69 (1.01)	0.446
	Head/neck (n = 48)	7.79 (1.53)	4.97 (1.54)	0.069
	Musculoskeletal/spine (n = 160)	11.73 (1.26)	7.89 (0.95)	0.024
	Superficial/minor (n = 170)	1.76 (0.51)	2.66 (0.66)	0.325
	Cardiac, vascular (n = 26)	8.84 (3.16)	10.00 (4.08)	0.749

**Figure 4:**
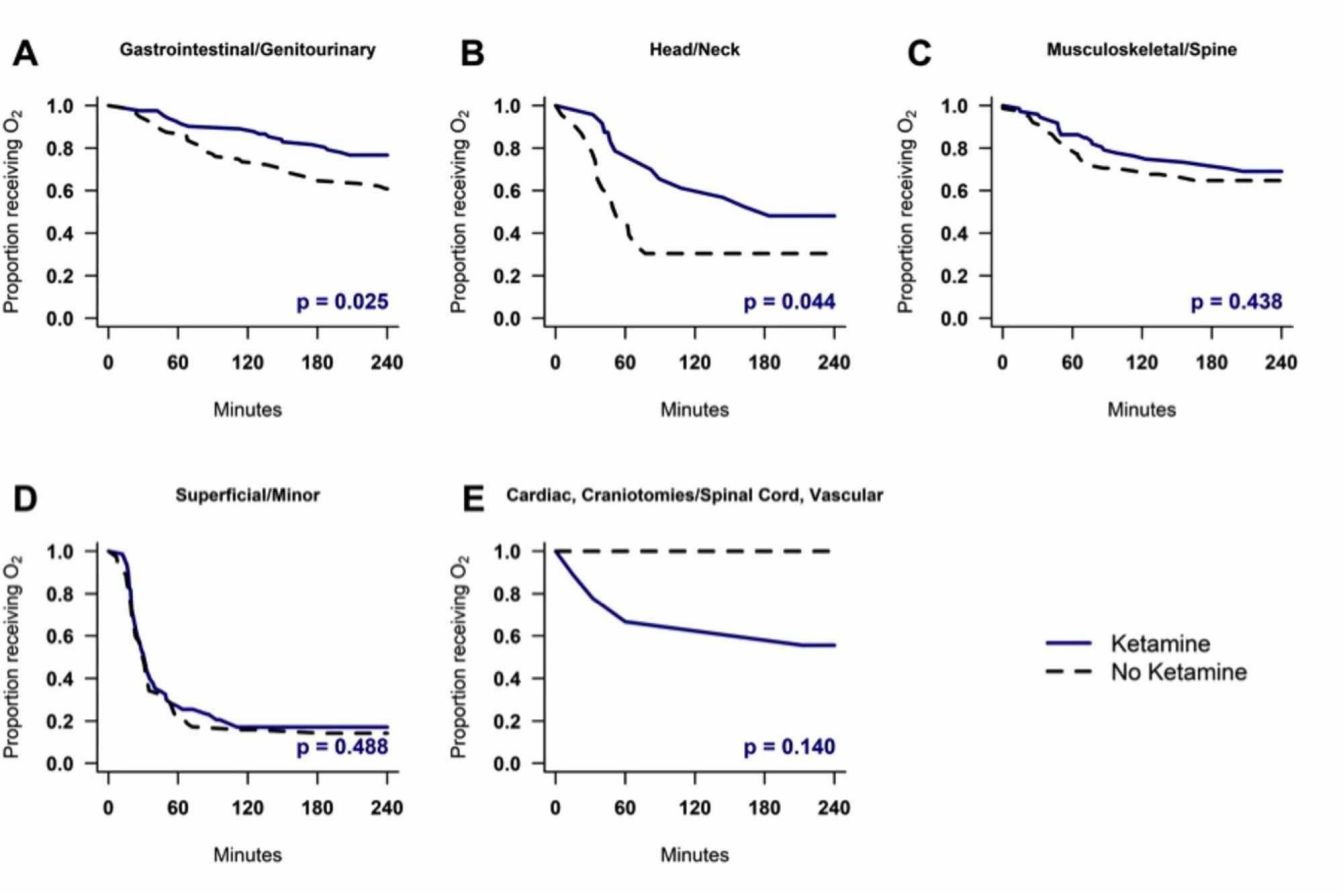
Time to wean to room air by surgery type.

Time to wean to room air was also significantly longer for ketamine patients compared with non-ketamine patients undergoing gastrointestinal/genitourinary (p = 0.025) and head/neck (0.044) surgeries (Figure 4).

## Discussion

In our study, low-dose intraoperative ketamine did not significantly decrease pain scores or OME requirements and did not improve standard respiratory recovery parameters for OSA patients after surgery. These results are in contrast to our hypothesis and are to previous findings in other studies of the general surgery population [11,13]. These conflicting results may be due to conditions specific to our study population involving only OSA patients or may be a result of clinical and/or methodological factors.

Though our findings were unexpected, further review of the published literature reveals a growing body of evidence that low-dose intraoperative ketamine may be ineffective in other populations as well. Cozowicz et al. published a retrospective study of 181,182 patients looking specifically at the use of multimodal analgesia in OSA patients who underwent elective joint arthroplasty [14]. In their multivariate analysis, COX-2 inhibitors and NSAIDs (non-steroidal anti-inflammatory drugs) were very effective in reducing post-operative opioid utilization; however, ketamine had no significant impact on opioid utilization. The 2017 PODCAST trial published in 2017 examined outcomes in 672 patients aged >60 years who received either 1.0 mg/kg ketamine, 0.5 mg/kg ketamine, or placebo intraoperatively [15], and found no difference in post-operative cognitive function, pain scores, or opioid consumption across the three groups.

In specific surgery populations, the value of low-dose ketamine has also been questioned. Moro et al. published a double-blind randomized clinical trial (RCT) of 135 patients undergoing laparoscopic cholecystectomy [16]. In comparing 0.4 mg/kg ketamine, 0.2 mg/kg ketamine, and control, they found no difference in the 40-question quality-of-recovery questionnaire, which includes time to eye opening, pain score, analgesic use, nausea, vomiting, and length of stay.

In an RCT of 80 open abdominal hysterectomy patients, Faiz et al. compared the efficacy of IV acetaminophen versus low-dose ketamine and found ketamine to be less effective for post-operative pain [17]. Aubrun et al. compared ketamine to placebo in a double-blind RCT of 90 hysterectomy patients and found no difference in analgesia, morphine consumption, or side effects [18]. Ganne et al. studied the effects of 0.15 mg/kg ketamine on induction followed by an infusion of 2 mcg/kg/minute ketamine in ear, nose, and throat surgery for cancer resection [19]. In their RCT of 62 patients, ketamine was no better than controls for VAS scores and morphine consumption up to 48 hours post-op. Engelhardt et al. used a slightly higher dose of 0.5 mg/kg ketamine and 4 mcg/kg/min infusion in his RCT of 34 pediatric scoliosis patients and also found no difference versus placebo in pain scores and morphine consumption up to 72 hours post-operative [20].

One theory for these findings in OSA patients is that ketamine contributes to sympathetic wind-up in OSA patients by blunting the inhibitory feedback to the central nervous system. In these patients who already have decreased pain tolerance relative to the general population [21], this could lead to increased pain perception post-operatively. Our findings also suggest that low-dose ketamine is ineffective in preserving their respiratory function, possibly due to the fact that their respiratory compromise is related to increased soft tissue causing airway compression rather than solely related to relaxation of airway musculature.

Lastly, current recommendations for dosing of ketamine use ideal body weight, but most prior studies are not focused on the use of ketamine in obese patients. Despite Gorlin et al.’s study showing that ketamine at doses < 0.3 mg/kg improved pain scores and opioid requirements [13], our results suggest that sub-anesthetic ketamine doses are inadequate to provide opioid-sparing effects in OSA patients. Mion demonstrated that following a bolus of 1 mg/kg ketamine, plasma concentration falls to below the analgesic threshold of 150 ng/mL after only 22 minutes [22]. Many studies demonstrating the efficacy of ketamine used continuous infusions through the surgery and even into PACU [11]. Very few of our patients received continuous ketamine infusions or higher anesthetic ketamine doses, which is generally accepted to be >1 mg/kg. However, based on our findings, side effects and significant respiratory depression in OSA patients could lead to increased post-operative complications rather than mitigating them in this high-risk population.

Possible confounders include the fact that ketamine is often used selectively for more painful surgeries, and despite matching this could still be an issue. Limitations of this study are those inherent to retrospective studies; data were not available for variables such as the severity of OSA, undiagnosed opioid abuse, and explicit drug or alcohol use, and for other psychosocial factors that could impact a patient’s pain perception in the post-operative period. Matching by intraoperative fentanyl dose also eliminates measurement of potential intraoperative opioid sparing, which is often considered in the measurements of prior studies. Also, ketamine patients typically received midazolam to avoid concern for dysphoria, and more sedating IV medications may have contributed to their increased oxygen needs post-operatively. ERAS (enhanced recovery after surgery) protocols and other multimodal analgesic use could mask the effects of ketamine. Lastly, although no difference in chronic opioid use was observed between the various groups, patients were not matched for chronic opioid use.

## Conclusions

Further research should be conducted regarding the use of ketamine in OSA patients. Large, randomized controlled trials in specific surgical populations could determine the utility, appropriate dosing, and timing of ketamine to definitively determine its beneficial effects, if any, in this high-risk patient population.
